# Immunomodulatory Activity and Protective Effects of Polysaccharide from *Eupatorium adenophorum* Leaf Extract on Highly Pathogenic H5N1 Influenza Infection

**DOI:** 10.1155/2013/194976

**Published:** 2013-09-18

**Authors:** Yi Jin, Yuewei Zhang, Chunyan Wan, Hongjun Wang, Lingyu Hou, Jianyu Chang, Kai Fan, Xiangming Xie

**Affiliations:** ^1^College of Biological Sciences and Biotechnology, Beijing Forestry University, 35 QingHua East Road, Beijing 100083, China; ^2^College of Veterinary Medicine, China Agricultural University, No. 2 Yuanmingyuan West Road, Beijing 100193, China

## Abstract

The development of novel broad-spectrum, antiviral agents against H5N1 infection is urgently needed. In this study, we evaluated the immunomodulatory activities and protective effect of *Eupatorium adenophorum* polysaccharide (EAP) against the highly pathogenic H5N1 subtype influenza virus. EAP treatment significantly increased the production of IL-6, TNF-**α**, and IFN-**γ** both *in vivo* and *in vitro* as measured by qPCR and ELISA. In a mouse infection model, intranasal administration of EAP at a dose of 25 mg/kg body weight prior to H5N1 viral challenge efficiently inhibited viral replication, decreased lung lesions, and increased survival rate. We further evaluated the innate immune recognition of EAP, as this process is regulated primarily Dectin-1 and mannose receptor (MR). These results indicate that EAP may have immunomodulatory properties and a potential prophylactic effect against H5N1 influenza infection. Our investigation suggests an alternative strategy for the development of novel antiinfluenza agents and benefits of *E. adenophorum* products.

## 1. Introduction

Highly pathogenic H5N1 subtype influenza virus can be transmitted directly from poultry to human and cause acute respiratory infections. Pandemic influenza virus H5N1 posed a worldwide threat to the public health because of rapid spread and high pathogenicity [1, 2]. The symptoms in animals or humans infected with H5N1 include fever, encephalitis, pneumonia, and severe acute respiratory syndrome (SARS) [3, 4]. The World Health Organization reported 622 human cases of highly pathogenic H5N1 influenza virus infection, including 371 deaths (a mortality rate >50%), from 2003 to 2013 (http://www.who.int/influenza/human_animal_interface/H5N1_cumulative_table_archives/en/index.html). Currently, the most effective preventive measure against the influenza virus is vaccination. Several antiinfluenza medications have been widely used, including zanamivir (Relenza) and oseltamivir (Tamiflu). Unfortunately, their benefits have been significantly restricted by drug-resistance and frequent antigenic mutation [5, 6]. Therefore, the development of novel antiinfluenza agents against the H5N1 subtype is very important.

The invasive plant *Eupatorium adenophorum,* native to Central America, has a strong ability to adapt to different environments all over the world. This plant first invaded southern Yunnan Province (China) in the 1940s from Burma and Vietnam, and quickly spread across southwestern China throughout the 1950s [7, 8]. Over the past 50 years, *E. adenophorum* has seriously impacted the ecological environment in China's middle subtropical zones, including Yunnan, Guizhou, Sichuan, and Guangxi Provinces, by encroaching farmlands, pasture fields, and forests [7]. Manual, chemical, or biological control of *E. adenophorum* has hindered its comprehensive development and utilization for economic benefit. Many bioactive components isolated from *E. adenophorum* have shown antimicrobial activity and immunomodulating properties [9]. In a recent study, the anti-inflammatory properties of ethanolic leaf extract was evaluated [10]. However, there have been few reports addressing the bioactivity of *E. adenophorum* polysaccharide (EAP).

The immunomodulating properties and therapeutic potential of a large number of botanical polysaccharides have been reported [11]. Several polysaccharides from *Cordyceps militaris*, *Portulaca oleracea, Gracilaria lemaneiformis, Gyrodinium impudium, *and* Panax ginseng* have been described as efficacious antiinfluenza agents against H1N1 and H3N2 strains [12–15]. In recent reports, polysaccharide-based adjuvants enhanced the immunogenicity and improved the protective efficacy of H5N1 vaccines in animal infection models [16, 17]. However, to our knowledge there have not been any reports regarding the treatment with EAP against highly pathogenic H5N1 influenza. 

In the present study, we investigated the potential effect of EAP against H5N1 influenza infection in a mouse model. Immune enhancement effects and the innate immune recognition of EAP were also evaluated. Our results suggest the anti-H5N1 effects of EAP offer an alternative strategy for developing antiinfluenza agents and the utilization of *E. adenophorum* products.

## 2. Methods

### 2.1. Virus

The H5N1 influenza virus (A/bar-headed goose/Qinghai/1/2010) used in this study was isolated from Qinghai Lake in May 2010. This isolate is highly pathogenic in poultry, mouse, and Madin-Darby canine kidney (MDCK) cells. The virus was propagated in MDCK cells at 37°C for 48 h, and the viral supernatant was harvested, aliquoted, and stored at −80°C. Viral titers were determined by plaque assay as described previously [18]. 

### 2.2. Animal and Cells

8–10-week-old Female BALB/c mice were obtained from Vital River Laboratories (Beijing, China), and the original breeding pairs were purchased from Charles River (Beijing, China). Mice were raised in independent ventilated cages (IVC) and received pathogen-free food and water. Animal treatments were governed by the Regulations of Experimental Animals of Beijing Authority, and approved by the Animal Ethics Committee of the China Agriculture University. 

The mouse leukemic monocyte macrophage Raw 264.7 cell line, human lung adenocarcinoma epithelial A549 cell line, and Madin-Darby canine kidney (MDCK) cell lines were provided by the Cell Resource Center of Peking Union Medical College. The cells were cultured and maintained according to the supplier's recommendations.

### 2.3. EAP Preparation

*E. adenophorum* was collected from Yunnan province, China. The leaves were sliced and dried in shade. 100 g dried materials were powdered in a mixer and then filtered with 40 meshes. Leaf powder was extracted by ultrasonic treatment with 1000 mL of distilled water for 45 min. The supernatant was collected and the precipitate resuspended in 1000 mL of distilled water and again extracted by ultrasonic treatment for 30 min. The resulting supernatant was combined with that obtained from the first ultrasonic treatment. The final aqueous fraction was evaporated to dryness in a rotary evaporator. The residue obtained was dissolved in distilled water and kept frozen at 4°C.

The extract was centrifuged at 3000 g/min for 25 min and concentrated under 80°C for 8 h to prepare polysaccharide. The supernatant was then deproteinized using the Sevag method, and dialyzed against water for 48 h. The final liquid was mixed with three-fold volume of 95% ethanol (v/v) and centrifuged at 3000 g/min for 10 min. The precipitates were successively washed with absolute ethanol, ether, and dried under vacuum at 40°C to obtained the crude polysaccharide (yield = 1.2%). EAP content was determined by the phenol-H_2_SO_4_ method [19].

### 2.4. Effects of EAP on Raw 264.7 Cells and A549 Cells *In Vitro*

2.5 mL A549 and Raw 264.7 cells (4 × 10^5^/mL) per well were plated in 6-well plates and cultured at 37°C under 5% CO_2_ for 24 h. Media was removed and 2.5 mL culture medium containing different concentrations of EAP (50, 100, 200 *μ*g/mL) was added to each well. Controls were treated with phosphate-buffered saline (PBS). Cells were collected 36 h after treatment for RNA extraction and quantitative polymerase chain reaction (qPCR). 

### 2.5. *In Vivo* Challenge Assay

Mice were administrated EAP at a dose of 5, 10, 25, or 50 mg/kg body weight, intranasally once daily for 5 days before the challenge. Control mice were administered PBS using the same schedule. Influenza virus stocks were diluted in PBS. Mice were anesthetized with Zotile (Virbac, France) intramuscularly at 15 mg/kg (body weight) and then infected intranasally with 120 plaque-forming units (PFU) of H5N1 influenza virus in 50 *μ*L. The lung tissue of five mice per group was collected on day 0 before challenge for qPCR and ELISA. Lung tissue from another five mice on day 3 postinfection was collected for plaque assay and qPCR. Ten mice per group were observed for survival for 14 days and body weights recorded.

### 2.6. Plaque Assay

MDCK cells were cultured in DMEM (Hyclone Laboratories, Logan, UT, USA) containing 10% FBS (Hyclone Laboratories), 100 U/mL penicillin, and 100 *μ*g/mL streptomycin (Invitrogen, San Diego, CA, USA). Lung tissue supernatant was diluted 10-fold and added to a cell monolayer covered by semisolid agar containing 0.5 *μ*g/mL of trypsin TPCK (Sigma-Aldrich, St. Louis, MO, USA). Plates were incubated at 37°C, 5% CO_2_ for 60–72 h and stained with 1% crystal violet.

### 2.7. RNA Extraction and qPCR

Total RNA from 1 × 10^6^ cells or 10 mg lung tissue were prepared by Trizol (Invitrogen) according to the manufacturer's instructions. DNaseI-treated RNA (0.2 *μ*g) was reverse transcribed into cDNA using random primers. The expression of the hemagglutinin (HA) gene of H5N1 influenza virus was detected by qPCR using the Power SYBR Green PCR Master Mix kit (Applied Biosystems, Foster City, CA, USA). The following primers were used: forward primer, 5′-CGC AGT ATT CAG AAG AAG CAAGAC-3′; and reverse primer, 5′-TCC ATA AGG ATA GAC CAG CTA CCA-3′. The reaction was run on an ABI 7500 thermal cycler with an initial denaturation step at 95°C for 10 min, followed by 40 cycles of 95°C for 15 s, 56°C for 30 s, and 72°C for 40 s. The copy number of the HA gene was calculated by 7500 software v2.0 (Applied Biosystems) using an HA-containing plasmid of known concentration as a standard.

Relative qPCR was performed for other eight genes: h*β*-actin, h IL-6, h IFN-*γ*, and hTNF-*α* for A549 cells; m*β*-actin, mTLR-2, mTLR-4, mDectin-1, mMR, mIL-6, mIFN-*γ*, and mTNF-*α* for Raw264.7 cells. The sequences of primers were shown in Table 1. The reaction was run with 95°C for 10 min, followed by 40 cycles of denaturation at 95°C for 15 sec, annealing at 52°C for 30 s, and extension at 72°C for 40 s. The fold change in gene expression was normalized to controls (naive mice) by 2^−ΔΔCT^ using **β**-actin as an internal standard [20].

### 2.8. ELISA

IL-6, TNF-*α*, and IFN-*γ* levels in lung were tested with ELISA kits (Boster, Wuhan, China) according to the manufacturer's protocol. One gram of lung tissue from each mouse was ground in 1 mL PBS and centrifuged for 20 min at 5000 rpm. The supernatants were collected and diluted 10-fold for ELISA.

### 2.9. Histopathological Analysis

Lung tissue was collected at day 3 postinfection and fixed by 4% neutral formalin at room temperature for 48 h. Serial tissue sections at 5-*μ*m-thick were obtained after embedding in paraffin. Each slide was examined by light microscopy (Olympus CX31, Center Valley, PA, USA) after staining with hematoxylin and eosin (H & E) or Masson trichrome (GenMed Scientifics Inc. Wilmington, DE, USA) then examined.

### 2.10. Statistical Analysis

The statistical analysis was performed using one-way ANOVAs with SPSS 12.0 (SPSS Taiwan Corp., Taiwan), and *P* < 0.05 was considered significant.

## 3. Results

### 3.1. Immunomodulatory Activities of EAP *In Vitro*

**Figure 1:**
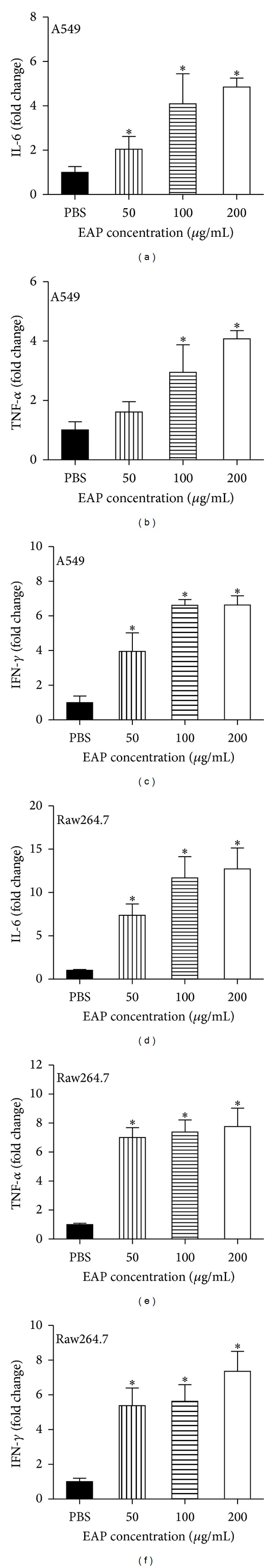
Effects of EAP on A549 and Raw264.7 cells. Cells were treated with various concentrations of EAP for 36 h. The mRNA levels of IL-6, TNF-*α*, and IFN-*γ* in A549 cells ((a)–(c)) and Raw264.7 cells ((d)–(f)) were determined by qPCR. *indicates *P* < 0.05 when compared with the PBS group.

Many botanical polysaccharides exhibit an immunomodulatory effect [11]. To determine the immunomodulatory properties of EAP, we investigated the potential effect of the polysaccharides on A549 and Raw264.7 cells. Cells were treated with various concentrations of EAP (50, 100, 200 *μ*g/mL) for 36 h. The mRNA levels of IL-6, TNF-*α*, and IFN-*γ* were detected by qPCR.  Figure 1 shows the immunomodulatory activities of EAP *in vitro*. Various concentrations of EAP triggered a strong secretion of IL-6, TNF-*α*, and IFN-*γ* in a dose-dependent manner both in A549 cells (Figures 1(a)–1(c)) and Raw264.7 cells (Figures 1(d)–1(f)) compared with the PBS treatment group.

### 3.2. Antiinfluenza Effect of EAP in H5N1 Infected Mice

**Figure 2:**
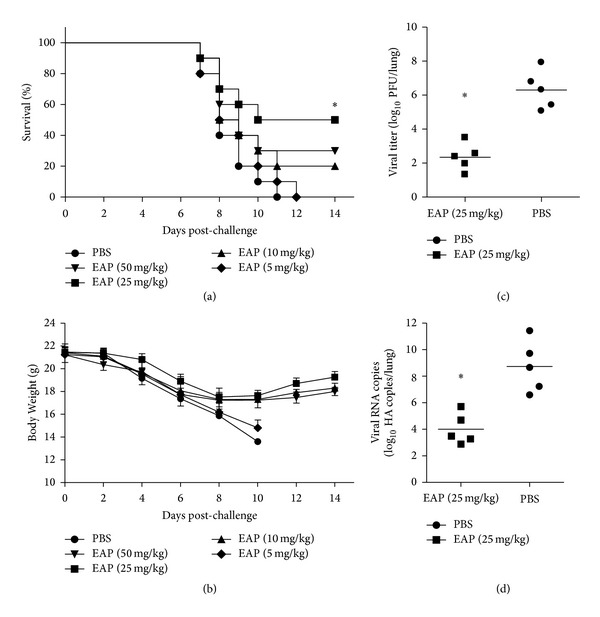
Effect of EAP on H5N1 infected mice. (a) Ten mice per group were monitored for 14 days for survival rate. The body weights were recorded (b). Lung viral load at day 3 postinfection was determined by plaque assay (c) and qPCR (d). *indicates *P* < 0.05 compared with the PBS group.

To test whether EAP could protect H5N1 infected mice, mice were treated with EAP at a dose of 5, 10, 25, or 50 mg/kg body weight intranasally once daily for 5 days prior to viral challenge with 120 PFU. Ten mice per group were monitored for 14 days for the survival rate. As shown in Figure 2(a), all mice receiving PBS died at day 11. Mice administrated 25 mg/kg EAP had a survival rate of 50% at day 14, which was significantly higher than those receiving PBS (by log rank analysis). EAP treatment of 10 mg/kg and 50 mg/kg also appeared to have a survival advantage, but not statistically significant. This result suggests that the protective effect of EAP against H5N1 infection requires a moderate dose. EAP treatment also alleviated weight loss in infected mice (Figure 2(b)).

 To determine the viral load in the lung of the infected mice, plaque assays and qPCR were performed. The pulmonary viral titers in the EAP (25 mg/kg) group were significantly lower than the titers in the mice that received PBS at day 3 postinfection (Figures 2(c) and 2(d)). These data clearly indicate that intranasal administration of EAP controls H5N1 viral replication and improves survival rates in a mouse model.

**Figure 3:**
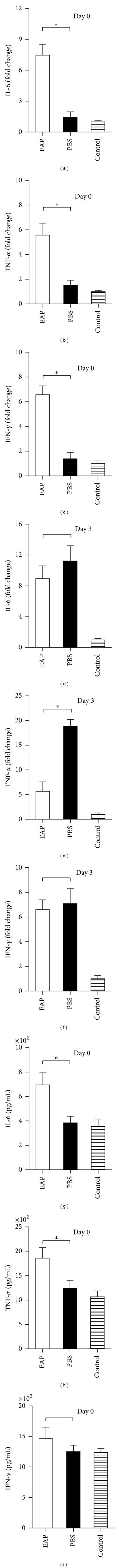
The expression of IL-6, TNF-*α*, and IFN-*γ  in vivo*. The expression level of IL-6, TNF-*α*, and IFN-*γ* in lung at day 0 was determined by qPCR ((a)–(c)) and ELISA ((g)–(i)). Day 3 postinfection levels were determined by qPCR ((d)–(f)). *indicates *P* < 0.05 compared with the PBS group.

The protective effect of EAP against H5N1 virus is likely due to its immunomodulatory properties. To detect IL-6, TNF-*α*, and IFN-*γ* expression, lungs of five mice per group were collected at day 0 before infection and tested by qPCR and ELISA. The mRNA levels in the EAP group (25 mg/kg) were significantly higher than those in the PBS control (naive mice) (Figures 3(a)–3(c)). Soluble cytokine levels at day 0 were measured by ELISA, and results were consistent with the qPCR results, even though IFN-*γ* production in the EAP group was not significantly higher than that of the PBS group (*P* = 0.0599) (Figures 3(g)–3(i)). These results suggest that EAP increases the IL-6, TNF-*α*, and IFN-*γ* production.

IL-6, TNF-*α*, and IFN-*γ* expression at day 3 postinfection was determined by qPCR. In contrast, TNF-*α* mRNA levels following EAP (25 mg/kg) treatment were significantly lower than those in the PBS group (Figure 3(e)), while IL-6 and IFN-*γ* expression were only slightly lower (not significant) (Figures 3(d) and 3(f)). These results may be explained by a higher viral load, and the more severe inflammatory response in PBS treated mice.

### 3.3. Histopathological Lesions in Lungs

**Figure 4:**
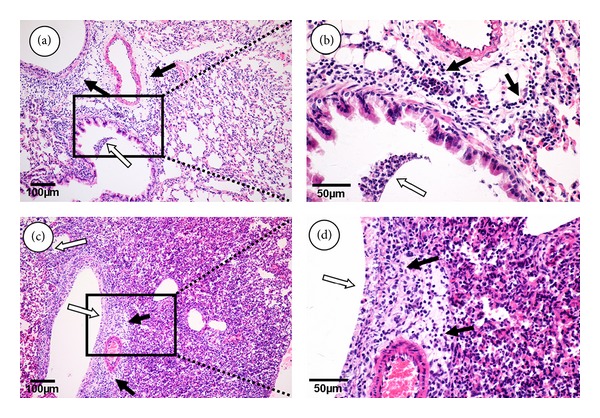
The lung histopathology of H5N1 infected mice. Representative lung sections from EAP group ((a), (b)) and PBS group ((c), (d)) at day 3 postinfection were stained with H & E. The solid arrows indicate interstitial edema and inflammatory cellular infiltration around small blood vessels. The unshaded arrows indicate a dropout of mucous epithelium in bronchioles and inflammatory cellular infiltration.

Excessive inflammation can cause severe lung lesions during H5N1 influenza infection. To evaluate histopathological changes in the lungs of infected mice, tissues of each group at day 3 postinfection were examined. The lungs of PBS treated mice exhibited a severe inflammation response, characterized by interstitial edema, inflammatory cellular infiltration around small blood vessels, alveolar lumen flooded with edema fluid mixed with exfoliated alveolar epithelial cells, and a thickening of alveolar walls (Figures 4(c) and 4(d)). The lungs of EAP (25 mg/kg) treated mice exhibited milder lesions than those receiving PBS, characterized by signs of bronchopneumonia with interstitial edema, and inflammatory cell infiltration around small blood vessels (Figures 4(a) and 4(b)). Viral loads and inflammatory cytokine production in the lung were correlated; suggesting that EAP treatment reduces lung lesions in H5N1 infected mice.

### 3.4. Innate Immune Recognition of EAP

**Figure 5:**
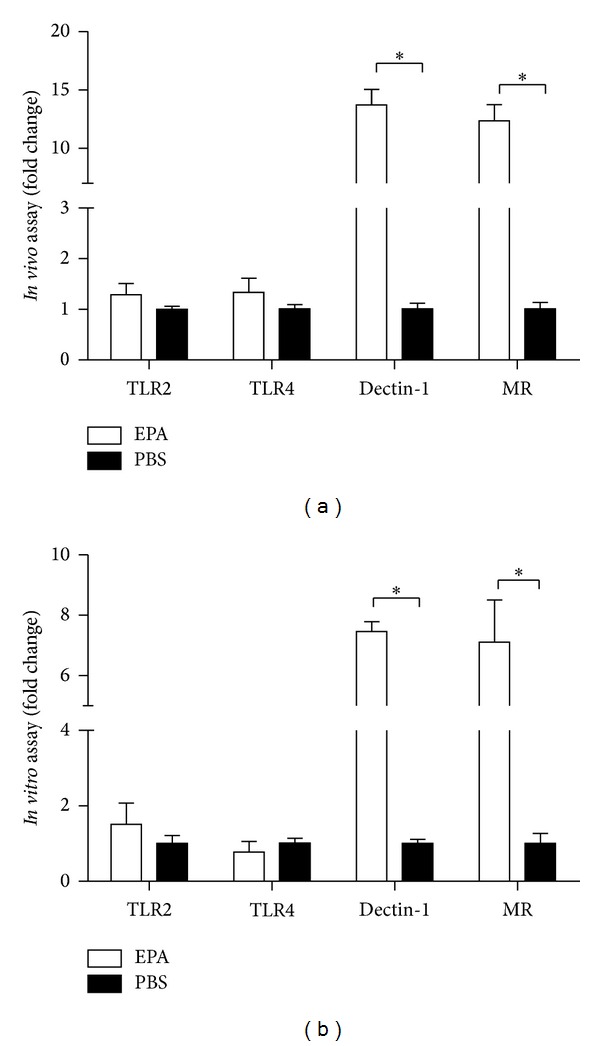
The expression of pattern recognition receptors. (a) Mice were treated with EAP (25 mg/kg body weight) intranasally once daily for 5 days while control mice received PBS. The TLR2, TLR4, Dectin-1, and MR mRNA levels in lung were detected by qPCR. (b) Raw264.7 cells were treated with 200 *μ*g/mL EPA for 36 h, and then qPCR. *indicates *P* < 0.05 compared with the PBS group.

Polysaccharides derived from many plants enhance the secretion of cytokines and chemokines, such as TNF-*α*, IL-6, IL-8, and IL-12 [11]. This immunomodulatory effect is mediated mainly through recognition of polysaccharide polymers by several pattern recognition receptors (PRRs). To determine which receptor contributes directly to the innate immune recognition of EAP, Toll-like receptor 2 (TLR2), TLR4, Dectin-1, and mannose receptor (MR) were examined by qPCR both *in vivo* and *in vitro*. Mice were treated with EAP at a dose of 25 mg/kg body weight intranasally once daily for 5 days, with control mice receiving PBS. Lung total RNA was prepared for qPCR. The expression of Dectin-1 and MR in EAP treated mice was significantly elevated compared with controls, while expression of TLR2 and TLR4 were slightly higher, but not statistically significant (Figure 5(a)). *In vitro* assay showed similar trends. As shown in Figure 5(b), Raw264.7 cells were treated with 200 *μ*g/mL EPA for 36 h before qPCR. Dectin-1 and MR levels were significantly higher, while expression of TLR2 and TLR4 did not change. These data suggest that EAP recognition occurred mainly via the Dectin-1 and MR pathway.

## 4. Discussion

In this study, we evaluated the immunomodulatory activities and protective effect of EAP against H5N1 influenza infection in a mouse model. To our knowledge, these findings are the first to show the anti-H5N1 effect of EAP. Intranasal administration of EAP prior to H5N1 viral challenge improved survival rates of infected mice with a corresponding reduction of pulmonary viral load. The anti-H5N1 effect was very likely due to the innate immune recognition of EAP and the secretion of innate immune mediators (IL-6, TNF-*α* and IFN-*γ*) before infection. Furthermore, the effect of EAP on PRR expression (including TLR2, TLR4, Dectin-1, and MR) was determined both *in vivo* and *in vitro*. These results suggest that the innate immune recognition of EAP was dependent upon the activation of the Dectin-1 and MR pathways. Our data demonstrate the feasibility of using EAP as a novel immunomodulatory agent against influenza infection. Unfortunately, the sugar composition of EAP has not been characterized. 

The emergence of new drug-resistant strains resulting from antigenic drift limits the therapeutic benefits of vaccination and antiviral agents in controlling influenza [6, 21, 22]. Thus, development of novel broad-spectrum antiinfluenza strategies is urgently needed. Most botanical polysaccharides are ideal candidates for novel immunomodulatory agents due to their nontoxic properties and fewer side effects compared with bacterially derived polysaccharides. A number of polysaccharides isolated from plant and fungi exhibit effective antiviral benefits against influenza A virus (including H1N1 and H3N2 subtypes) [12–15]. The use of polysaccharides as immunomodulatory agent in anti-H5N1 studies is rare. In this paper, our data show the immunomodulatory activities of EAP both *in vivo* and *in vitro*. EAP treatment elevated the production of IL-6, TNF-*α*, and IFN-*γ* and provides a survival advantage in H5N1 infected mice. The survival rate following EAP pretreatment (25 mg/kg body weight) was significantly higher than in mice receiving PBS (50% to 0%). 

In previous reports, high levels of proinflammatory cytokines and chemokines (including TNF-*α*, IL-6 and IFN-*γ*) were detected during H5N1 infection [23, 24]. This “cytokine storm” leads to the severe respiratory symptoms and host immune injury. Thus, H5N1-induced cytokine storms are hypothesized to be the main cause of mortality, and the use of anti-inflammatory agents may therefore provide a therapeutic effect [25, 26]. However, it is unclear whether the lack of proinflammatory cytokines (such as TNF-*α* and IL-6) facilitates viral clearance. Interestingly, knockout mice deficient in TNF-*α*, TNF-*α* receptor, IL-6, MIP-1*α*, and IL-1R or steroid-treated, wild-type mice did not have a survival advantage compared with wild-type mice following H5N1 influenza infection [27, 28]. Interestingly, prophylactic treatment of TLR3 agonist PolyICLC, which strongly upregulates cytokine production, provides protection against H1N1 and H5N1 infections [29, 30]. These conflicting studies may be explained in that the inflammatory response helps clear the virus, while aggravating host pathological damage. Elevated production of cytokines, such as IL-6, TNF-*α*, and IFN-*γ* are very important for viral clearance in the early stage of infection by activating the innate immune system. Once the viral infection has triggered a cytokine storm due to the high viral load, the inflammatory response causes severe pathological injury or even death. In this case, receiving an immunomodulator alone cannot help animal to survive [25]. This likely explains why immunomodulator treatment prior to viral infection results in a better survival rate [26, 30]. In our study, treatment of EAP shortly after infection or 24 h postinfection did not provide a survival advantage (data not show). 

The antiinfluenza properties of IL-6, TNF-*α*, and IFN-*γ* have been discussed in many studies, despite their participation in cytokine storms triggered by influenza infection. IL-6 plays an important role in protecting against influenza A virus as it is required for viral clearance and essential for animal survival [31]. TNF-*α* has been reported to exert a defensive effect against influenza infection *in vitro* [32]. IFN-*γ* treatment in the early stages of influenza infection improves the survival rate in mouse models [33]. In addition, high levels of IFN-*γ* secretion stimulated by ginseng polysaccharides provide an antiinfluenza effect *in vivo* [12]. In this report, intranasal administration of EAP before H5N1 challenge elevates expression of IL-6, TNF-*α*, and IFN-*γ* compared with mice receiving PBS. The high levels of these mediators contribute to the viral clearance and antiviral response. Pulmonary viral titers following EAP treatment were lower at day 3 postinfection. In contrast, IL-6 and IFN-*γ* mRNA levels were slightly lower, while TNF-*α* production was significantly lower than that of PBS group. Regarding the excessive inflammation induced by H5N1 virus, massive secretion of mediators contributes to lung injury rather than an antiviral response. Therefore, the timing of EAP treatment as a prophylactic agent is very important.

The immunomodulatory activities of botanical polysaccharides are thought to be mediated by several PRRs [11]. In this study, we examined the mRNA levels of TLR2, TLR4, Dectin-1, and MR after EAP treatment. EAP was found to upregulate Dectin-1 and MR mRNA expressions significantly both *in vivo* and* in vitro*. Our hypothesis is that the innate immune recognition of EAP is driven mainly via a Dectin-1 and MR dependent pathway. Binding to these receptors, EAP may activate complex intracellular signaling pathways, and increase cytokine production, leading to an antiviral response. Thus, the protection against H5N1 by EAP treatment is less likely to cause drug resistance, and may represent a broad-spectrum antiinfluenza effect.

In conclusion, our study demonstrates that EAP leaf extract is a prophylactic and immune enhancement agent against H5N1 influenza virus infection. Treatment with EAP effectively inhibits H5N1 viral replication and improves animal survival. This approach offers an alternative strategy for antiinfluenza immunomodulatory agent development, and benefits the utilization of *E. adenophorum* products.

## Figures and Tables

**Table 1:** The sequences of qPCR primers used in this study.

Primers	Sequences	
	Forward primer	Reverse primer
h*β*-actin	5′-GTG GGG CGC CCC AGG CAC CA-3′	5′-CTC CTT AAT GTC ACG CAC GAT TTC-3′
h IL-6	5′-CCT TCG GTC CAG TTG CCT TCT-3′	5′-CCA GTG CCT CTT TGC TGC TTT C-3′
h IFN-*γ*	5′-TCC AAC GCA AAG CAA T-3′	5′-CAG GCA GGA CAA CCA T-3′
hTNF-*α*	5′-GAG TGA CAA GCC TGT AGC CCA TGT TGT AGC-3′	5′-GCA ATG ATC CC A AAG TAG ACC TGC CCA GAC-3′
m*β*-actin	5′-GAG ACC TTC AAC ACC CCG C-3′	5′-ATG TCA CGC ACG ATT TCC C-3′
mTLR-2	5′-GTC ACT ATC CGG AGG TTG CAT-3′	5′-CAA CAC CTC CAG CGT CTG AG-3′
mTLR-4	5′-TTC TTC TCC TGC CTG ACA CCA-3′	5′-TTA AAT TCT CCC AAG ATC AAC CGA T-3′
mDectin-1	5′-GCC CTT GTC CTC CTA ATT GGA-3′	5′-CCC AGT TGC CAG CAT TGT CT-3′
mMR	5′-TCT TGG GTC GGA TGA TTC TG-3′	5′-GCC TGC TCT TCC TCT GAC CT-3′
mIL-6	5′-AGC CAG AGT CCT TCA-3′	5′-TCT TGG TCC TTA GCC-3′
mIFN-*γ*	5′-AGT GGC ATA GAT GTG GAA-3′	5′-GAC CTG TGG GTT GTT GA-3′
mTNF-*α*	5′-GGG TGT TCA TCC ATT CTC-3′	5′-GGA AAG CCC ATT TGA GT-3′
